# Hominem Sine Opus Spatium: Where Do the Ideas Come from to Move the Brain, Mind, Behaviour and Neurosciences in Malaysia?

**DOI:** 10.21315/mjms2018.25.2.1

**Published:** 2018-04-27

**Authors:** Jafri Malin Abdullah

**Affiliations:** Malaysian Journal of Medical Sciences, Universiti Sains Malaysia Health Campus, 16150 Kubang Kerian, Kelantan, Malaysia

**Keywords:** brain, mind, behaviour, psychiatry, neurosciences, Malaysia

## Abstract

People can work wonders without a room. Rooms make people think within a box, and people who are not confined within a room can wonder while thinking and solve problems as they see them in the environment. The dearth in the growth of professionals trained in the neurosciences who will use neurotechnology in the future is a dire situation facing Malaysia, according to the Academy of Sciences Malaysia’s 2017 Emerging Science, Engineering and Technology (ESET) study. Further, this human resource needs to be fundamentally cultivated at schools from a very young age. The author describes the activities that have taken place in the country via a bottom-up approach over the last two years and hopes that eventually these endeavours will end with the creation of an ASEAN Brain, Mind, Behaviour and Neuroscience Institute for Creativity and Innovation being established with the full support of the Government of Malaysia or other local and international financial donors.

Since the last Malaysian Journal of Medical Sciences (MJMS) editorial at the end of 2015 (1), which discussed improvements in the neurosciences in terms of research, creativity and innovation, Neuronman aka the author has focussed on activities that promote the brain, mind and neurosciences in the field of academia, research and clinical fields, and these activities have grown tremendously.

Modern humans are psychologically required to have a room or roof over their heads before they can individually or as a group plan, prepare, implement and sustain activities (2–13). The activities that are described below, however, were performed by individuals not requiring a dedicated physical and personal indoor space, and I am grateful to Almighty Allah (SWT) for allowing the team to work together in order to produce such a great quantity of work in the field of brain, mind and neurosciences and to make them become a reality.

The Joint Integrated Clinical Psychology courses, which are three new courses of study for a Master of Psychology (Clinical Psychology), Doctorate of Psychology (Clinical Psychology) or Doctorate of Psychology (Clinical Neuropsychology), a joint degree between Universiti Sains Malaysia (USM) and Universiti Pendidikan Sultan Idris (UPSI) (Figure 1), were approved on 12 October 2017 along with 1a 1b a Master of Cognitive Neurosciences on 22 February 2018. The second degrees is given in collaboration with seven schools at the Universiti Sains Malaysia which includes the Schools of Medical Sciences, Educational Sciences, Humanities, Management, Graduate Schools of Business, Social Sciences and Computer Sciences along with the Institute of Postgraduate Studies (Figure 2). The overseer for these four courses is the School of Medical Sciences, Universiti Sains Malaysia Health Campus. We hope to use USM@ Kuala Lumpur to teach these course works in the field of Psychology and Cognitive Neurosciences.

Recently, on 28 July 2017, the Brain, Mind and Neuroscience Research Foundation was formed with eight founding members, namely Professor Dato’ Dr Jafri Malin Abdullah (Universiti Sains Malaysia), Associate Professor Dr Durriyyah Sharifah Hasan Adli (Universiti Malaya), Associate Professor Datuk Dr Mohamed Saufi Awang (International Islamic University Malaysia), Dr Ashraf Sharifuddin (Hospital Sultanah Aminah), Dr Nujaimin Udin (Hospital Sultanah Nur Zahirah), Dr Nasser Abdul Wahab (Hospital Pulau Pinang), Dr Mohd Sofan Zenian (Hospital Queen Elizabeth, Kota Kinabalu), and Dr Sim Sze Kiat (Universiti Malaysia Sarawak & Hospital Umum Sarawak) (Figure 3) with the purpose of promoting research in Malaysia in this field.

We congratulate the national and zone coordinators as we open the sixth year of the Malaysian Brain Bee Competition in order to attend the International Brain Bee Competition. We also congratulate the tenth batch of new students of the Integrated Postgraduate Neuroscience Program joining in 2018 at USM (Figure 4). We are also proud to announce that the International Youth Neuroscience Association, USA (Figure 5) in early 2018 selected Malay College Kuala Kangsar to be the Malaysian Chapter representative for all 18 neuroscience clubs in Malaysia in order to coordinate their activities. This is the seventh year after the first neuroscience clubs were established in Sekolah Menengah Sains Tengku Muhammad Faris Petra, Kota Bharu, Kelantan, and Sekolah Kubang Kerian 3, Kota Bharu, Kelantan.

The Vacation Research Program, running in its sixth year, has accepted the largest number of students, who will be exposed to neuroscience STEM lab projects for a duration of six to nine weeks (Figure 6). Most of these students will go on to take up medicine or science-based courses for their undergraduate programs.

We were honoured to be involved in preparing two sub-documents involving Foresight 2050 for the Government of Malaysia via the Academy of Medicine. These two documents, entitled Emerging Science, Engineering and Technology (ESET) Study and Envisioning Malaysia in 2050: A Foresight Narrative with their weblink: https://issuu.com/asmpub/docs/eset_study_report and https://issuu.com/asmpub/docs/envisioning_malaysia_2050_foresight, respectively looked at numerous technologies, one of which was neurotechnology, and their future development towards the year 2050. The launch of the Science and Technology Foresight Malaysia 2050: Emerging Science, Engineering and Technology (ESET) Study was held on 12 October 2017 at 10:00 am in conjunction with the launch of the National Innovation and Creative Economy Expo 2017 (NICE’17) at Technology Park Malaysia by YAB Dato’ Seri Dr Ahmad Zahid Hamidi, Deputy Prime Minister of Malaysia (14).

The second report is Envisioning Malaysia in 2050: A Foresight Narrative by the Academy of Sciences Malaysia. The study, which was initiated in 2015, was conceived as a guiding framework for a bold journey between Malaysia’s status quo and its future in the year 2050 and was prepared by the Academy of Sciences Malaysia (ASM) in collaboration with Ministry of Sciences, Technology and Innovation (MOSTI). The report was launched by the Minister of Science, Technology and Innovation, YB Datuk Seri Panglima Wilfred Madius Tangau, on 2 November 2017 during the ASEAN 2050 Forum: Fourth Industrial Revolution that took place from 2–3 November 2017 at the Putrajaya Marriot Hotel (Figure 7) (15).

We hope that with all the emphasis on cognitive neurosciences, neuroimaging and neurotechnology especially with endevours to bring neurosciences closer to psychology (Figure 8) and our center’s initiation of mascots (Figure 9); Malaysia will be able to initiate a Brain, Mind and Neurosciences initiative that will make it as one important focus in the ASEAN region. At the current moment, the Center for Neuroscience Services and Research, Universiti Sains Malaysia is focussed on storing in our servors; International Neuroinformatic Coordinating Facility (INCF) “friendly” raw and pre-processed data with the assistance of Hospital USM and School of Computer Sciences, USM as well as with the assistance of the INCF Malaysian node coordinator (Figure 10).

At least this man without a room better known as Neuronman is proud to have trained to date 65 brain, spine and nerve surgeons for the country (Figure 11) and hope all that is planned will achieve success for the university and the country. 7a 7b

## Figures and Tables

**Figure 1a–1c:**
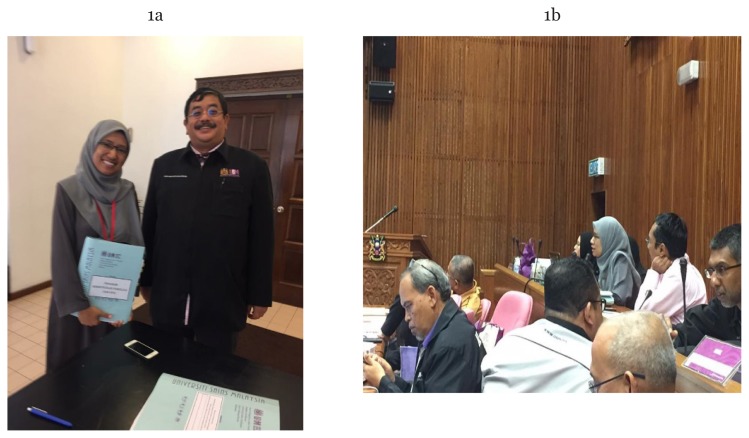

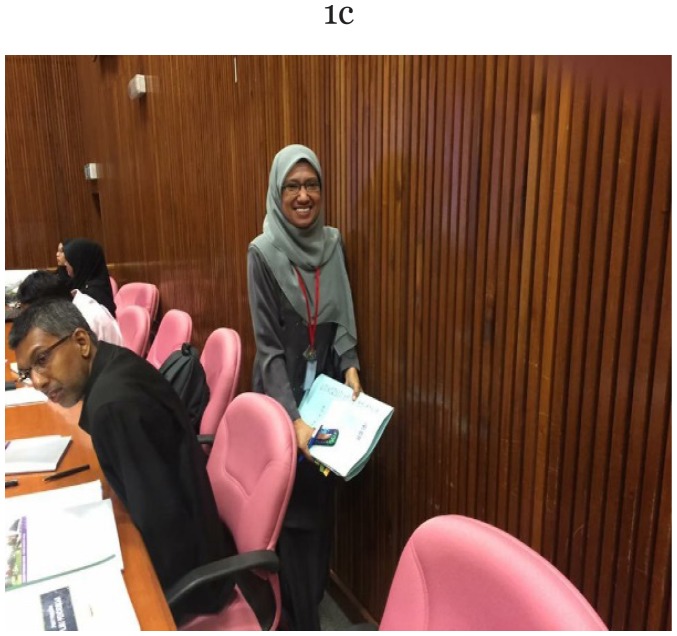
The defense of the Joint Integrated Clinical Psychology Program USM-UPSI at the senate Universiti Sains Malaysia was done by Associate Professor Dr Azizah Othman and Professor Dato’ Dr Jafri Malin Abdullah on 24 May 2017

**Figure 1d:**
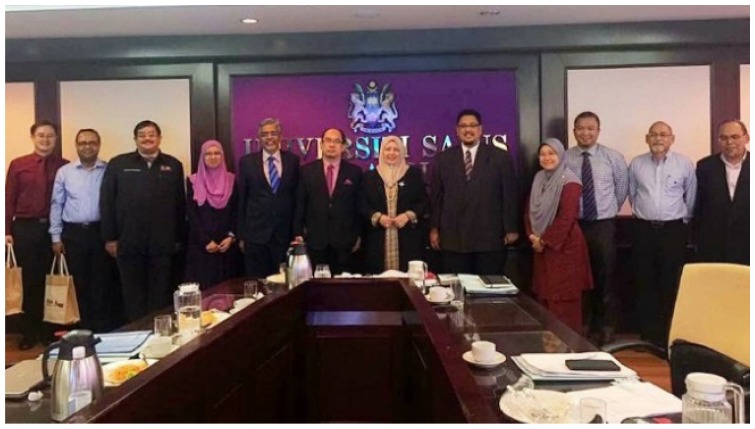
Board of Studies for Joint Integrated Clinical Psychology courses, on 19 May 2017 at UPSI, attended by the Vice Chancellor of Universiti Sains Malaysia and Universiti Pendidikan Sultan Idris. From the left: Associate Professor Dr Alvin Ng Lai Oon, Associate Professor Dr Shamsul Haque, Professor Dato’ Dr Jafri Malin Abdullah, Associate Professor Dr Azizah Othman, Professor Rahmatullah Khan Abdul Wahab Khan, Professor Dato’ Dr Mohammad Shatar Sabran, Professor Datuk Dr Asma Ismail, Professor Dr Ahmad Farhan Mohd Sadullah, Professor Dr Siti Raudzah Ghazali, Professor Dr Shaiful Bahari Ismail, Professor Dr Rozman bin Din and Associate Professor Dr Ahmad Mohamad

**Figure 1e:**
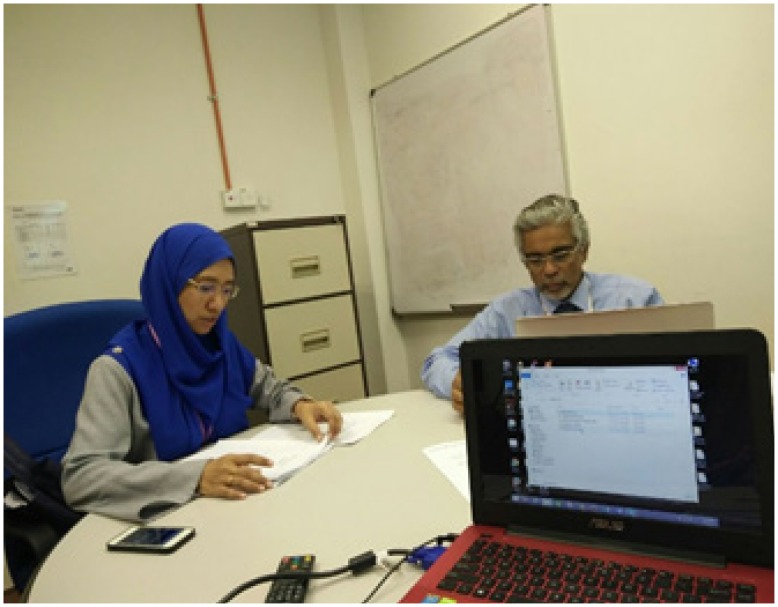
Associate Profesor Dr Azizah Othman from Department of Pediatrics, School of Medical Sciences, Universiti Sains Malaysia and Professor Rahmatullah Khan Abdul Wahab Khan from Faculty of Human Development, Universiti Pendidikan Sultan Idris (UPSI) are pictured discussing about joint psychology programme

**Figure 1f:**
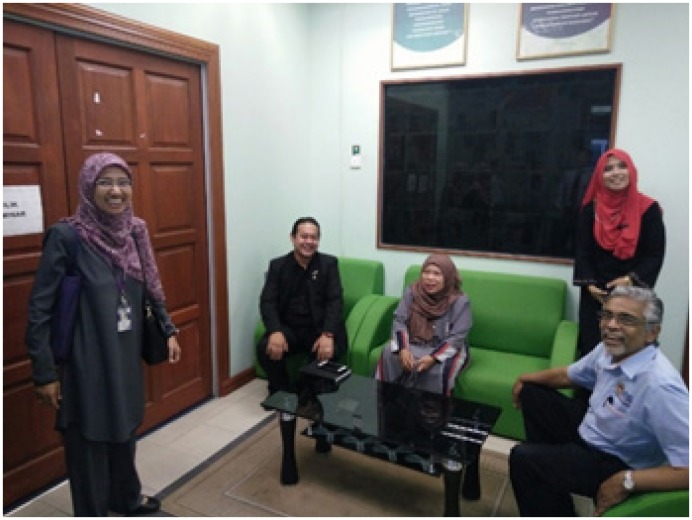
Mdm Wan Nur Fajrina Wan Azmi (Senior Assistant Registrar), Associate Professor Dr Azizah Othman (Coordinator Joint Integrated Clinical Psychology USM) being visited by Professor Dr Siti Eshah binti Mokshein (Dean), Mr Suhaimi Zulkipli (Assistant Registrar), and Professor Dr Rahmatullah Khan Abd Wahab Khan (Coordinator Joint Integrated Clinical Psychology UPSI), from Faculty of Human Development, Universiti Pendidikan Sultan Idris (UPSI) at Universiti Sains Malaysia, Health Campus during a visit to the Universiti Sains Malaysia Health Campus on the 19 September 2017 scrutinising the clinical wards and clinics at Hospital USM as well as the teaching and learning facilities for postgraduate students in the School of Medical Sciences, USM. This is the first clinical psychology postgraduate programme in Malaysia that was initiated between a neurosurgeon, Professor Dato’ Dr Jafri Malin Abdullah and a clinical psychologist, Professor Dr Rahmatullah Khan leading to three postgraduate courses co-organised by the School of Medical Sciences that is joint and integrated

**Figure 2a:**
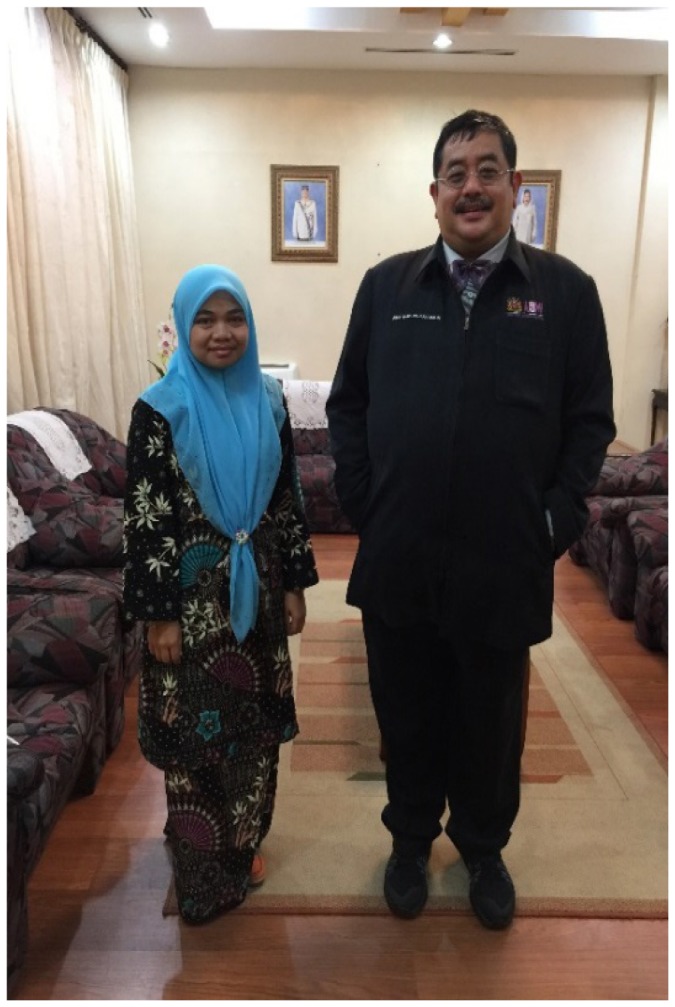
Professor Dato’ Dr Jafri Malin Abdullah with Dr Aini Ismafairus Abd Hamid who developed the course and defended the coursework during senate Universiti Sains Malaysia on 4 Disember 2017 after the Board of Studies on the 6 September 2017

**Figure. 2b:**
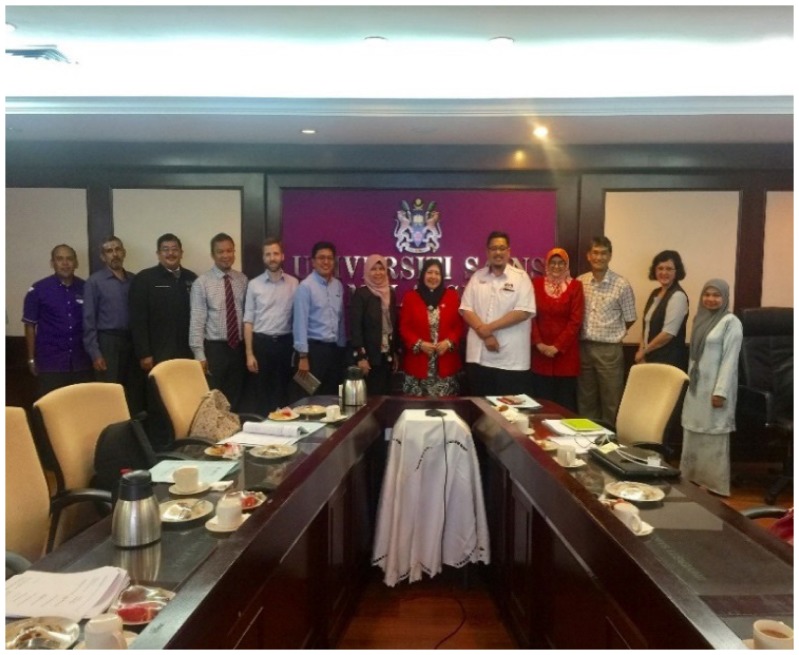
From the left: Associate Professor Dr Ahmad Mohamad, Professor Dr Ahamad Tajudin Khader, Professor Dato’ Dr Jafri Malin Abdullah, Professor Dr Shaiful Bahari Ismail, Associate Professor Dr Alexandre Scharfer, Encik Rushdi Abdul Rahim, Associate Professor Dr Norsiah Fauzan, Professor Datuk Dr Asma Ismail, Professor Dr Ahmad Farhan Mohd Sadullah, Professor Dr Habibah Abdul Wahab, Professor Dr Mohd Nazalan Mohd Najimudin, Professor Dr Azlinda Azman and Dr Aini Ismafairus Abd Hamid after Board of Studies for Master in Cognitive Neuroscience at Universiti Sains Malaysia on 6 September 2017

**Figure 2c:**
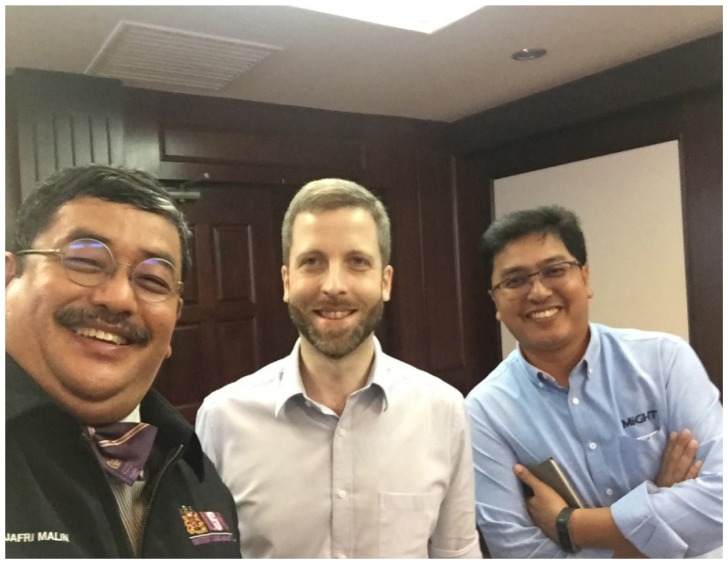
From the left: Professor Dato’ Dr Jafri Malin Abdullah with Associate Professor Dr Alexandre Scharfer and Mr Rushdi Abdul Rahim

**Figure 2d:**
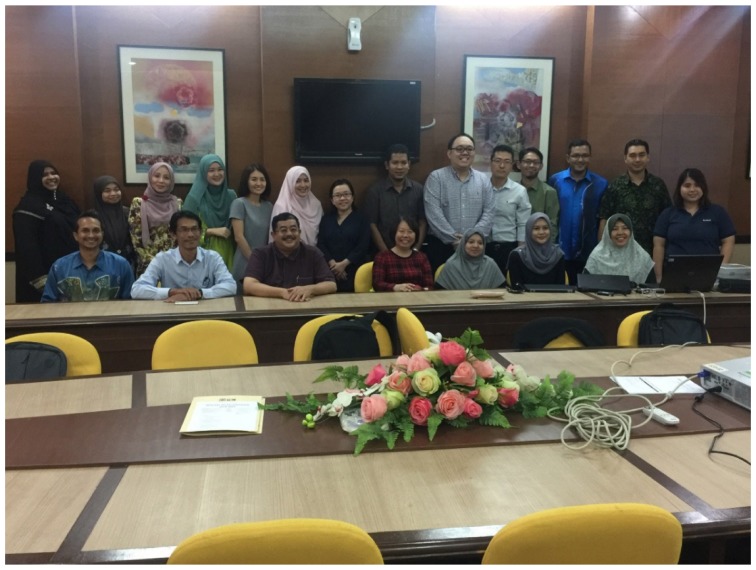
Group photo of lecturers involved in Master of Cognitive Neuroscience course on the 29 November 2017 until 1 Disember 2017. From the left, seated: Associate Professor Dr Aznan Che Ahmad, Associate Professor Dr Putra Sumari, Professor Dato’ Dr Jafri Malin Abdullah, Professor Dr Lee Lay Wah, Dr Nor Azila Noh, Nur Shahirah Md Nor, Associate Professor Dr Aswati Hamzah. From the left, standing: Dr Nurul Hashimah Ahamed Hassain Malim, Dr Aini Ismafairus Abd Hamid, Dr Salmiza Saleh, Dr Shaizatulaqma Kamalul Ariffin, Dr Chin Phaik Nie, Dr Salmi Mohd Isa, Dr Low Hui Min, Mr Hazim Omar, Mr Daniel Chan (DanMedik Sdn Bhd Company), Dr Fan Jie (DanMedik Sdn Bhd Company), Dr Mohamed Faiz Mohamed Mustafar, Associate Professor Dr Haidi Ibrahim, Dr Paramjit Singh Jamir Singh and Ms Chiew Yinn (DanMedik Sdn Bhd Company)

**Figure 3:**
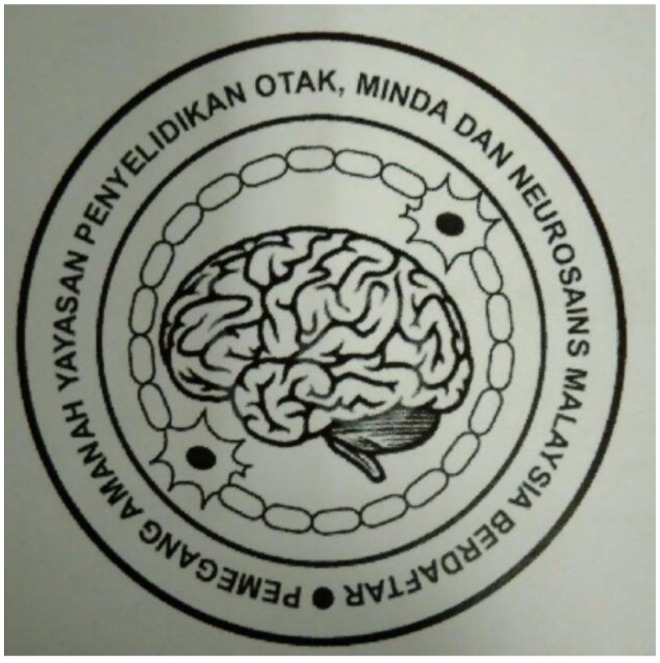
The logo of the newly established Malaysian Brain, Mind and Neuroscience Research Foundation which was registered using it’s Bahasa Malaysia translation

**Figure 4:**
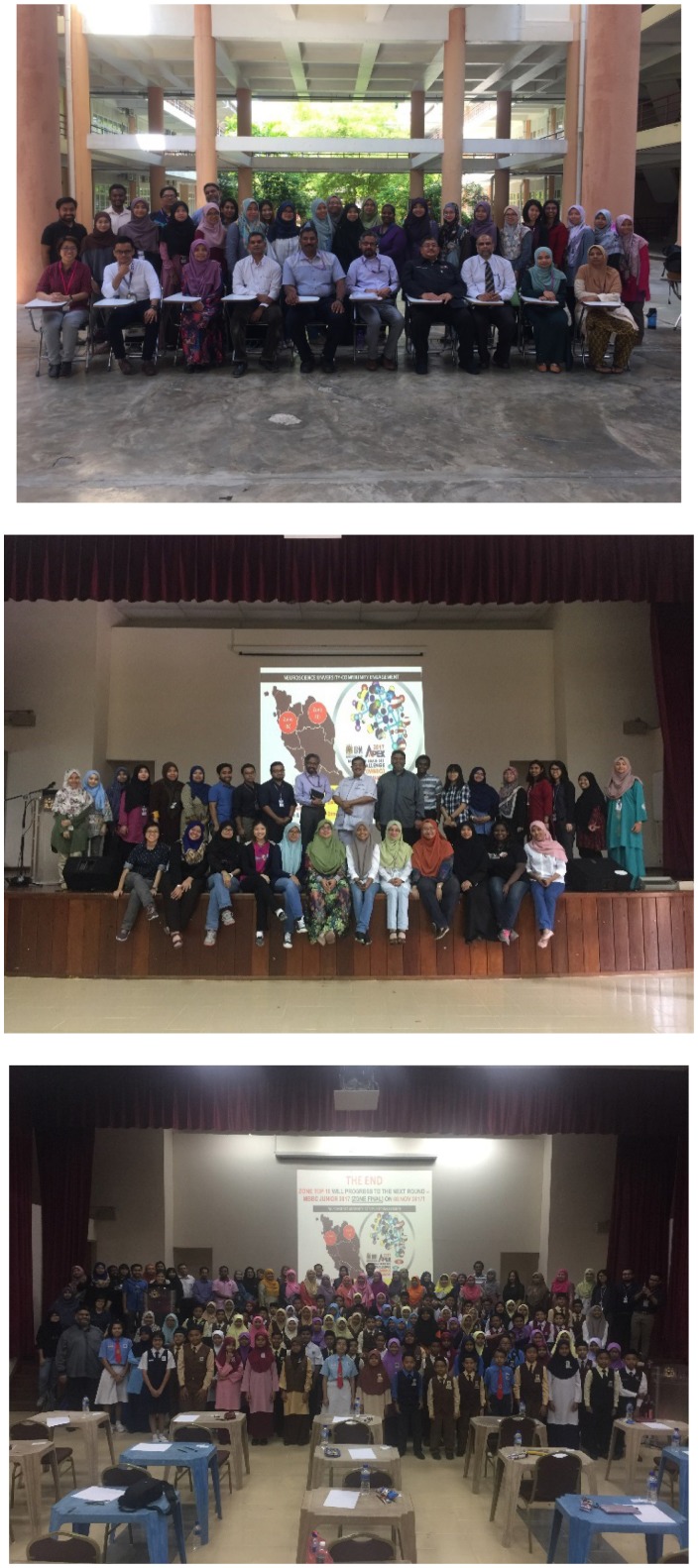
Group photographs of some of the lecturers and 9th Batch INP students involved in the 10th Batch of Integrated Neuroscience Programme (INP) student orientation session on the 11 Februari 2018 after the Junior Malaysian Brain Bee Competition 2017

**Figure 5a & 5b:**
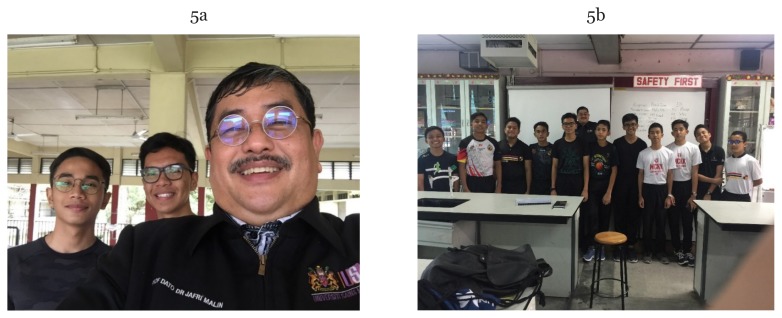
The Malay College Kuala Kangsar Neuroscience Club has became the coordinator of the Malaysia Chapter of the International Youth Neuroscience Association headed by Alexander Skvortsov, Dr Norbert Myslinski and Professor Dato’ Dr Jafri Malin Abdullah

**Figure 5c:**
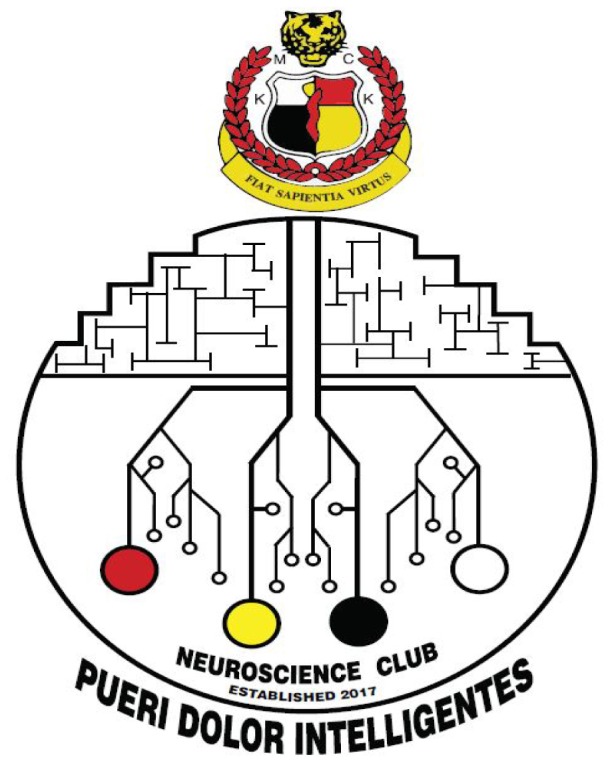
The logo for Malay College Kuala Kangsar Neuroscience Club

**Figure 5d:**
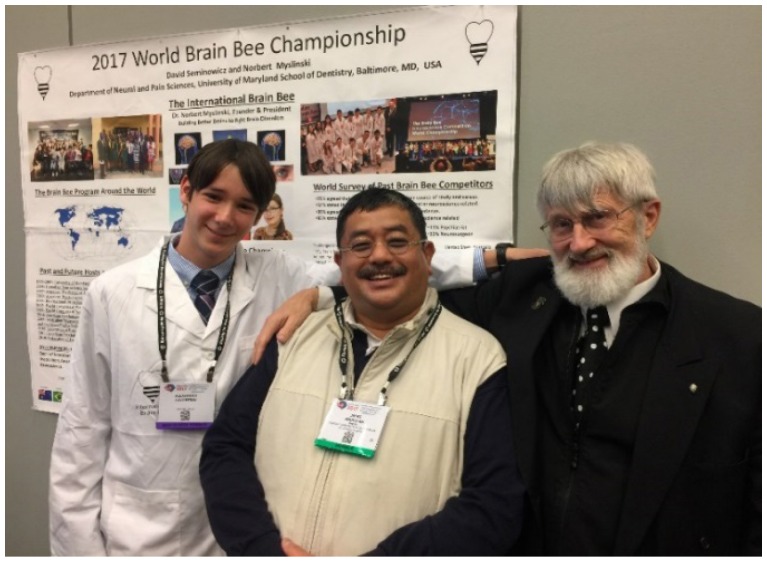
Professor Dato’ Dr Jafri Malin Abdullah with Alexander Skvortsov and Dr Norbert Myslinski

**Figure 5e:**
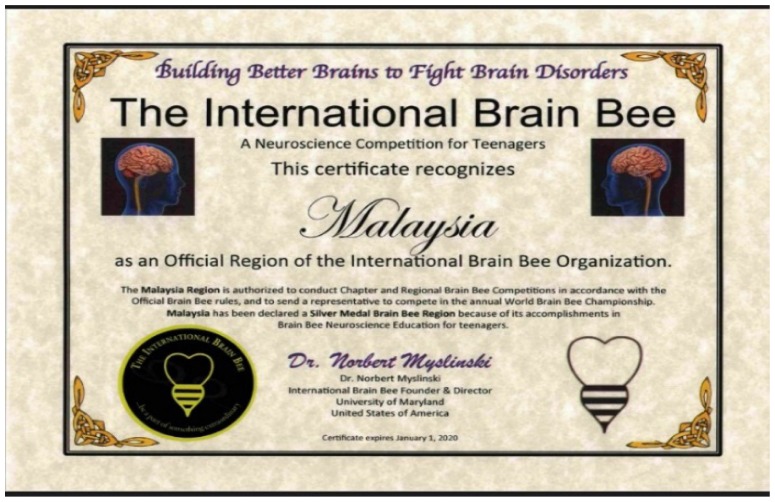
Certificate that indicates Malaysia as an official region of the International Brain Bee Organization

**Figure 6a–6f:**
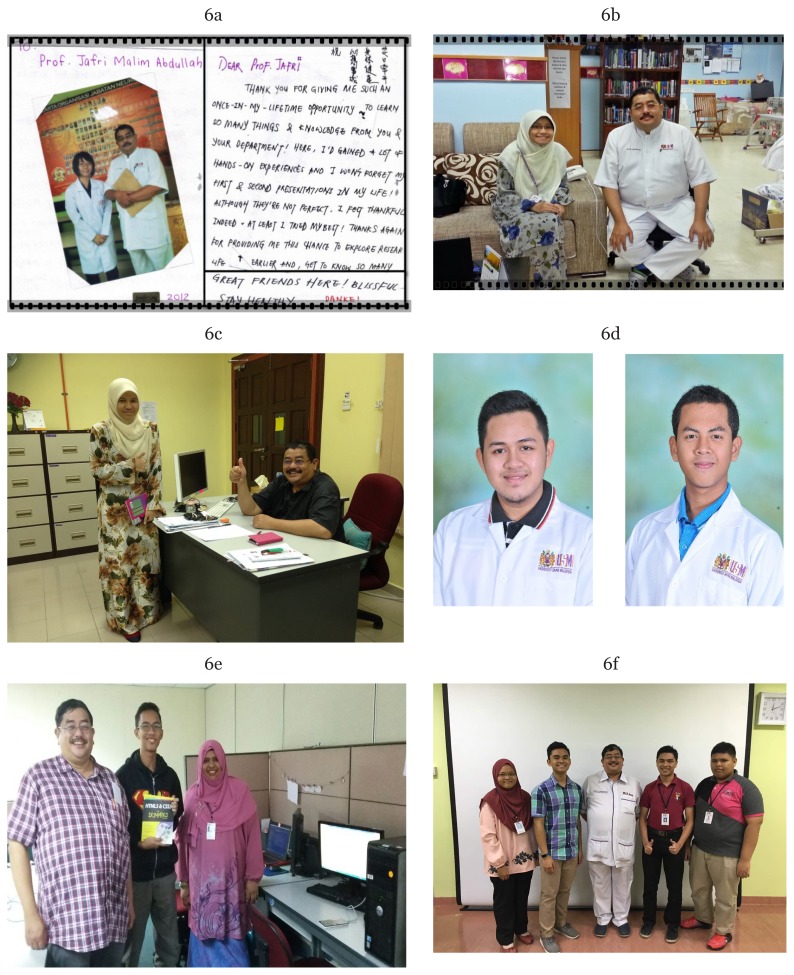
Vacation Research Programme has begun seven years ago with the involvement of numerous lecturers from the Department of Neurosciences, School of Medical Sciences, Health Campus, Universiti Sains Malaysia, School of Computer Sciences and Centre for Drug Research, Main Campus, Universiti Sains Malaysia

**Figure 7a & 7b:**
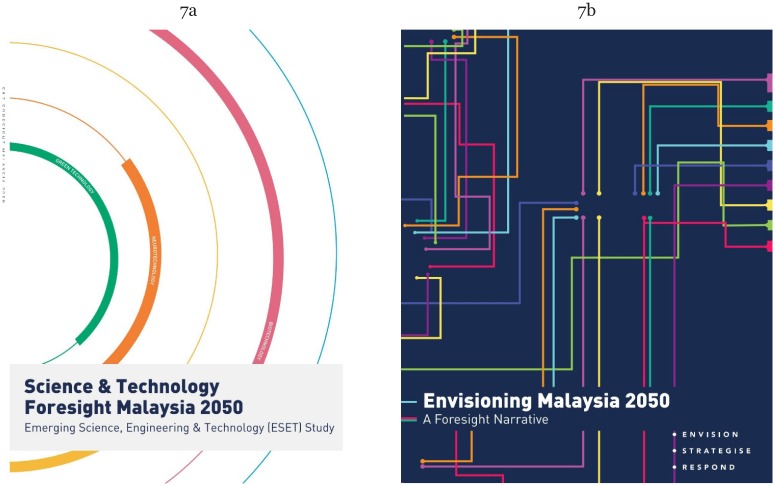
Both two books with sub-chapter on neurotechnology have been produced by Academy Science Malaysia 2017 to highlight the needs for cognitive neuroscience and neuroimaging in all aspect of Malaysia Life towards the year 2050

**Figure 8:**
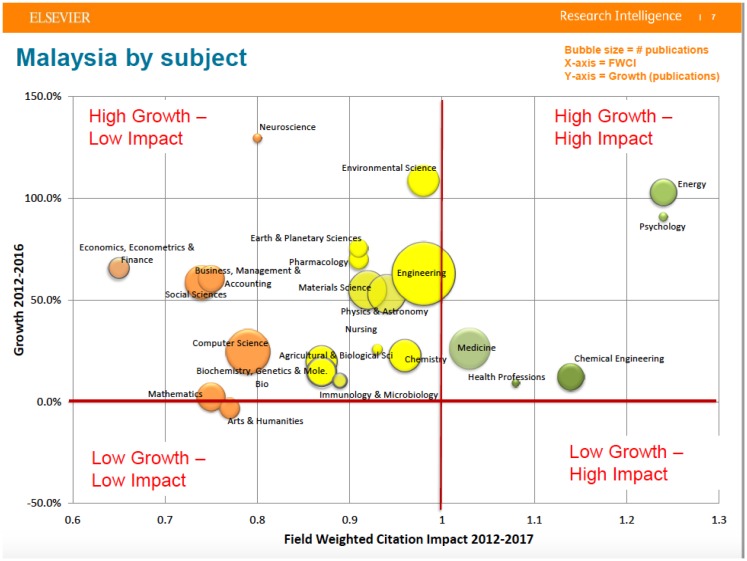
Neuroscience in Malaysia has combined with Psychology via the Department of Neurosciences, School of Medical Sciences, Universiti Sains Malaysia and the Center of Neuroscience Services and Research, Universiti Sains Malaysia to be High Growth High Impact

**Figure 9a:**
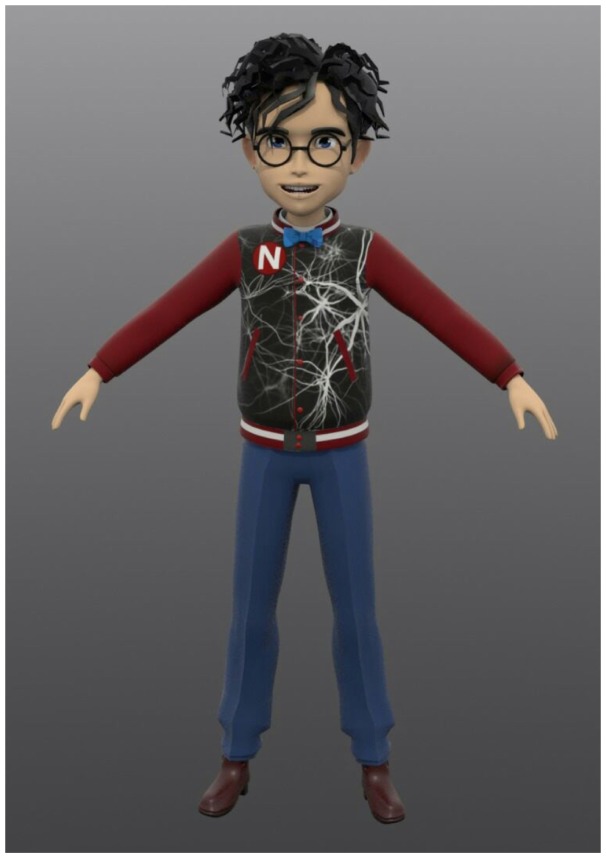
Neuron Man was created initially as a mascot for P3Neuro (Centre for Neuroscience Services & Research), Universiti Sains Malaysia and now used as a trademark and copyright

**Figure 9b:**
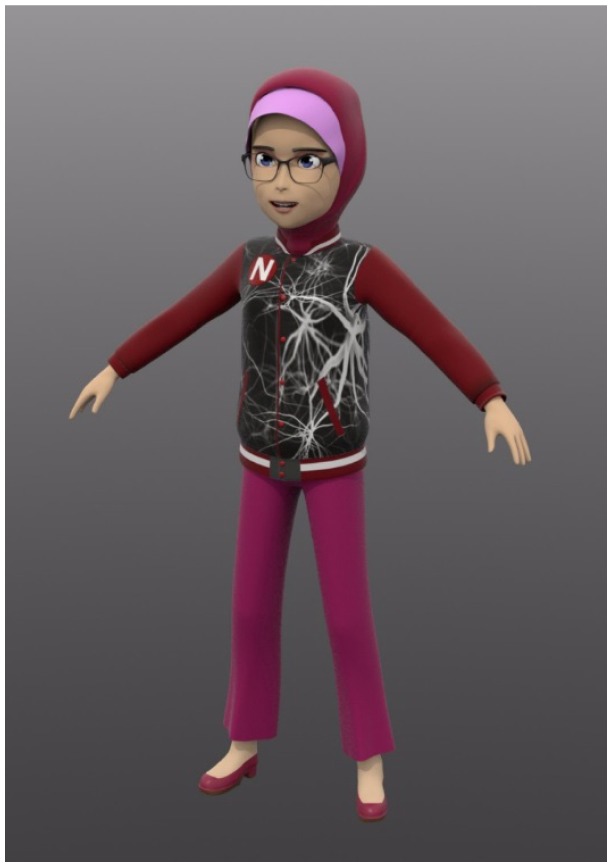
Neuron Woman was created initially as a mascot for P3Neuro (Centre for Neuroscience Services & Research), Universiti Sains Malaysia in 2017 and now used as a trademark and copyright

**Figure 9c:**
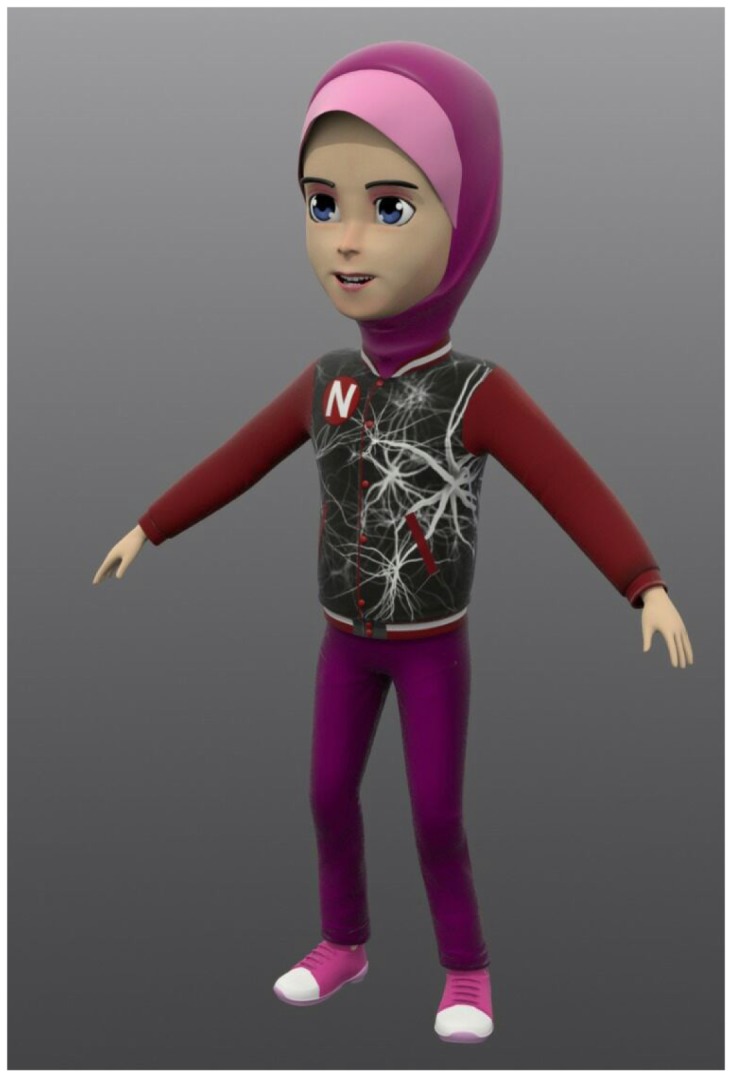
Neuron Girl was created initially as a mascot for P3Neuro (Centre for Neuroscience Services & Research), Universiti Sains Malaysia in 2017 and now used as a trademark and copyright

**Figure 9d:**
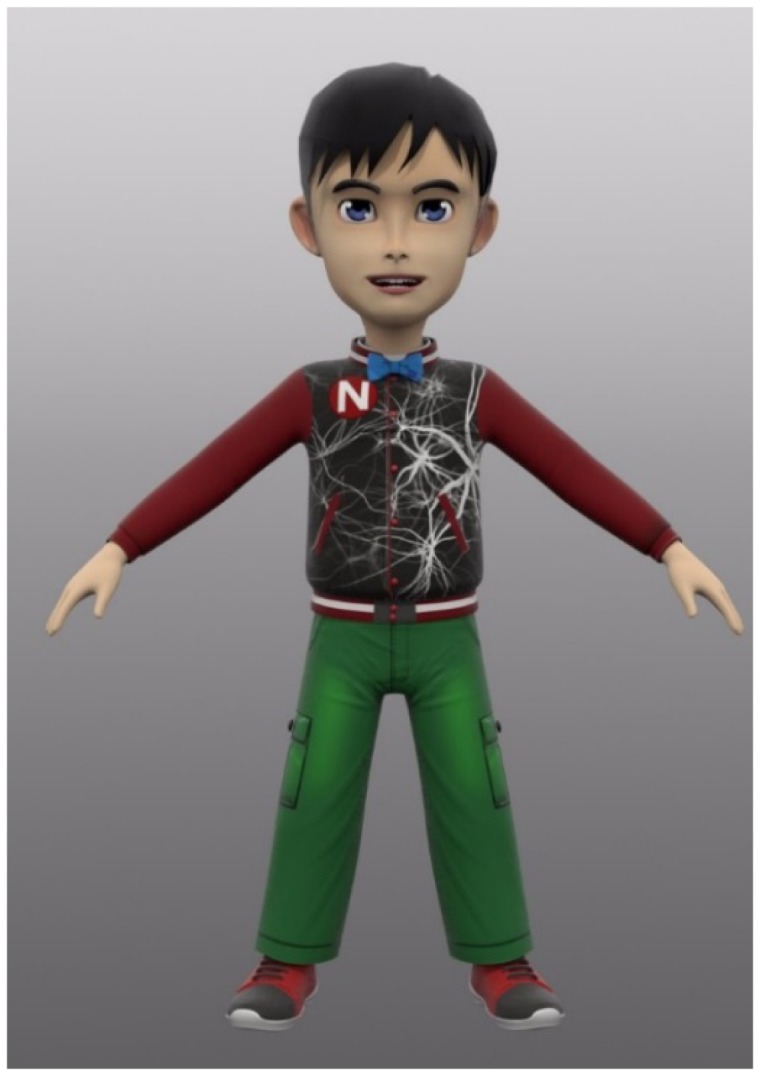
Neuron Boy was created initially as a mascot for P3Neuro (Centre for Neuroscience Services & Research), Universiti Sains Malaysia in 2017 and now used as a trademark and copyright

**Figure 9e:**
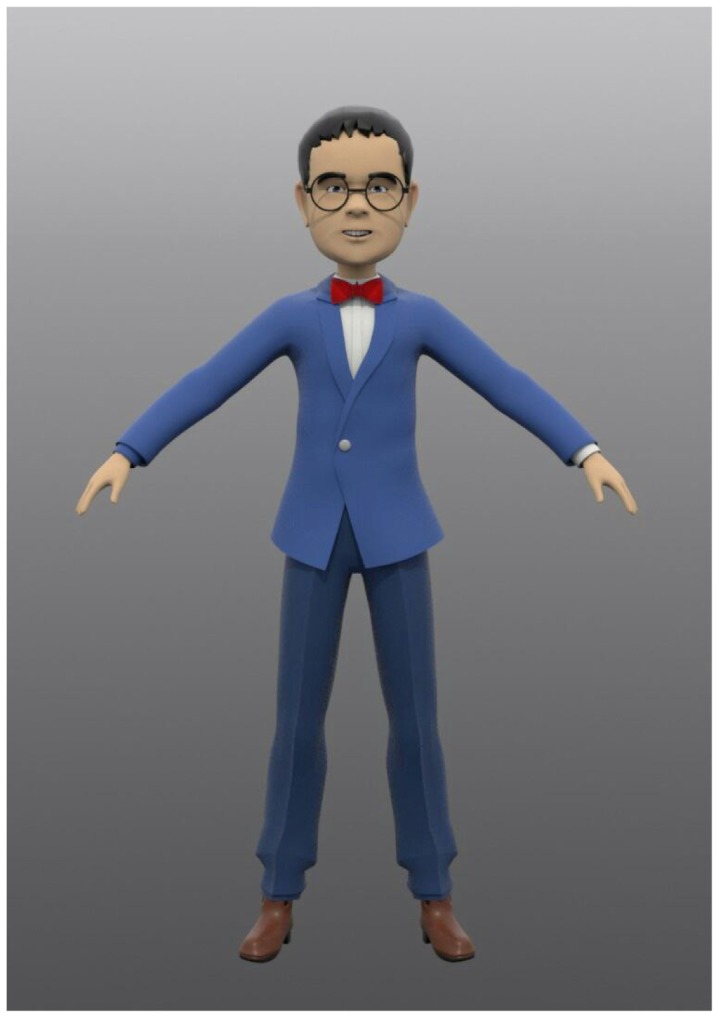
Neuron–Gardener Sifoo was created initially as a mascot for P3Neuro (Centre for Neuroscience Services & Research), Universiti Sains Malaysia in 2017 and now used as a trademark and copyright

**Figure 9f:**
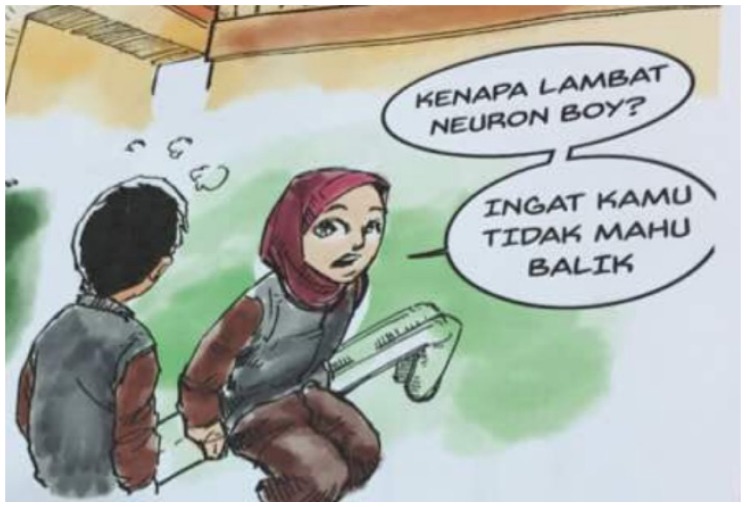
These characters were used for a STEM Comic Project of the Ministry of Education in 2018

**Figure 10a–10c:**
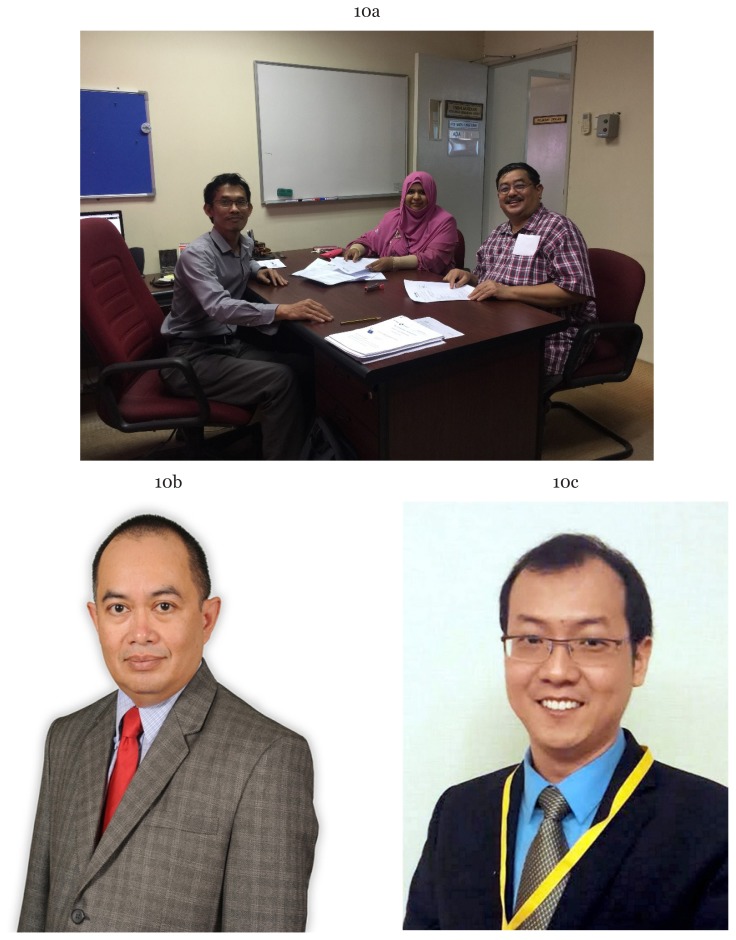
The collaborative efforts of Associate Professor Dr Putra Sumari, Dr Nurul Hashimah Ahamed Hassain Malim and Dr Ariffin Marzuki Mokhtar from Hospital Universiti Sains Malaysia, Dr Eric Ho Tatt Wei, INCF Malaysia Node representative to push Neuro Data Storage for Malaysia via the Center for Neuroscience Services and Research, USM (P3Neuro) begins February 2018
